# Phenolic Antioxidants in Aerial Parts of Wild *Vaccinium* Species: Towards Pharmaceutical and Biological Properties

**DOI:** 10.3390/antiox8120649

**Published:** 2019-12-16

**Authors:** Oana-Crina Bujor, Corneliu Tanase, Mona Elena Popa

**Affiliations:** 1 Research Center for Studies of Food Quality and Agricultural Products, University of Agronomic Sciences and Veterinary Medicine of Bucharest, Marasti Blvd. No. 59, RO-011464 Bucharest, Romania; oana.bujor@qlab.usamv.ro; 2 Faculty of Pharmacy, “George Emil Palade” University of Medicine, Pharmacy, Sciences and Technology of Târgu-Mures, Gh. Marinescu Street No. 38, RO-540139 Tîrgu Mures, Romania; 3 Faculty of Biotechnology, University of Agronomic Sciences and Veterinary Medicine of Bucharest, Marasti Blvd. No. 59, RO-011464 Bucharest, Romania; monapopa@agral.usamv.ro

**Keywords:** polyphenols, antioxidants, *Vaccinium*, leaves, stems, natural resources, pharmaceutical products

## Abstract

Phenolic compounds are a widespread group of secondary metabolites found in all plants, representing the most desirable antioxidants due to their potential to be used as additives in the food industry (inhibition of lipid oxidation), and in cosmetology and medicine (protection against oxidative stress). In recent years, demand for the identification of edible sources rich in phenolic antioxidants, as well as the development of new natural plant products to be used as dietary supplements or pharmaceuticals, has been a great preoccupation. At present, from the “circular economy” perspective, there is an increased interest to use agricultural waste resources to produce high-value compounds. *Vaccinium* leaves and stems are considered essentially an agro-waste of the berry industry. Scientific studies have shown that phenolic compounds were found in a markedly higher content in the leaves and stems of *Vaccinium* plants than in the fruits, in agreement with the strongest biological and antioxidant activities displayed by these aerial parts compared to fruits. This paper aims to review the current state of the art regarding the phenolic antioxidants from leaves and stems of two wild *Vaccinium* species, bilberry (*Vaccinium myrtillus* L.) and lingonberry (*Vaccinium vitis-idaea* L.), as promising natural resources with pharmaceutical and biological activity.

## 1. Introduction

The interest in phenolic compounds has grown over recent years, particularly because they are excellent antioxidants. Consumption of antioxidants has shown its efficiency in the prevention of cancer, cardiovascular diseases, osteoporosis, obesity, diabetes, and skin aging [1]. The antioxidant properties of plant phenolic compounds are relevant in the field of food (inhibition of lipid oxidation), physiology (protection against oxidative stress), and cosmetology. They reflect the UV filter and reducing properties of these compounds and their ability to interact with metal ions and proteins [2]. In particular, phenolic compounds provide antioxidant activity by directly reducing reactive oxygen species (ROS), inhibiting enzymes involved in oxidative stress, binding metal ions responsible for the production of ROS, and stimulating endogenous antioxidant defense systems [3].

The quality and quantity of phenolic compounds in plants are generally influenced by the stage of growth, the parts of the plant to be used, the environmental growing conditions (temperature, sunlight, soil nutrients, the latitude and altitude of the location where the plants are growing, geographical locations) and genetic factors [4,5,6,7,8]. As described by Jovančević et al. [9], the total phenolic content of bilberry harvested from localities exposed to the sun was higher compared with plants grown in shadow. In the same study, it was shown that at an altitude higher than 1500 m, the amount of total phenolics is higher. Scientific studies have shown that *Vaccinium* species are excellent sources of phenolic compounds that are recognized for their bioactive value. Bilberry (*Vaccinium myrtillus* L.) and lingonberry (*Vaccinium vitis-idaea* L.) are the most significant wild species of the *Vaccinium* genus whose aerial parts (leaves and stems) are known as natural sources of food, beverages, dietary supplements, and pharmaceutical products due to their richness in nutritional and bioactive phenolic compounds [10,11,12,13,14]. In addition, *Vaccinium* berries are now the main plant parts used commercially, whereas their leaves and stems are considered essentially a waste byproduct of the berry industry [11]. At the same time, comparative studies on the phenolic composition of bilberry and lingonberry showed that phenolic compounds were found in a markedly higher content in the leaves and stems than in the fruits, in agreement with the strongest antioxidant capacity displayed by leaves and stems compared to fruits [10,15]. Furthermore, leaf and stem extracts of bilberry and lingonberry were found to protect dietary lipids from oxidation in an in vitro model of gastric digestion [16]. Increased attention for these raw materials is associated with their phenolic composition, antioxidant activity, and potential health-related benefits. The high phenolic content found in leaves and stems of *Vaccinium* plants is thought to be linked to their biological functions.

This review reports a comprehensive study, leading to the pharmaceutical and biological activities of phenolic secondary metabolites isolated from the leaves and stems of *Vaccinium* plants, with focus on the wild species bilberry (*Vaccinium myrtillus* L.) and lingonberry (*Vaccinium vitis-idaea* L.).

## 2. Phenolic Composition of Aerial Parts of *Vaccinium myrtillus* L. and *Vaccinium vitis-idaea* L.

The main classes of phenolic compounds present in the leaves and stems of *Vaccinium myrtillus* L. and *Vaccinium vitis-idaea* L. are phenolic acids (mainly chlorogenic acid), flavonoids, flavonol glycosides, and (epi)catechin monomers and oligomers [6,10,12,14,15,17,18], all known to be powerful antioxidants that act by directly trapping ROS, chelating transition metal ions, and inhibiting enzymes involved in the oxidative stress [19,20]. The recent studies conducted by Bujor et al. [10,15] showed that phenolic compounds were found in significantly higher contents in the leaves and stems than in the fruits. In *Vaccinium myrtillus* L. leaves, caffeic acid derivatives were the most representative group of phenolic compounds, with levels ranging between 67 and 79% of the dry extract (DE) weight, whereas in their stems, flavanol oligomers were the major group, representing between 54 and 62% of the total phenolic content [10]. As far as *Vaccinium vitis-idaea* L. is concerned, in leaves like in stems, flavanol oligomers were found to be the most abundant class of phenolic compounds, with relative levels ranging from 36 to 48% and 47 to 50%, respectively.

A summary of the studies of literature regarding the various phenolic compounds identified in *Vaccinium myrtillus* L. and *Vaccinium vitis-idaea* L. aerial parts is shown in Table 1.

## 3. Extraction and Separation of *Vaccinium* Phenolic Compounds

Due to the structural diversity and complexity of the phenolic compound, extraction is the first and the most important step in the separation of these compounds. The most common liquid/liquid and solid/liquid extractions are frequently employed to separate phenolic compounds. The phenolic nature of polyphenols makes them relatively hydrophilic, thus free phenolic compounds, including aglycones, glycosides, and oligomers, are extracted using water, polar organic solvents such as ethyl acetate, methanol, ethanol, acetonitrile, acetone, and their mixture with water or non-polar solvents (chloroform, diethyl ether) [24]. 

At present, regarding the overall environmental impact of industrial extraction, the concept of green extraction has been introduced to protect both the environment and consumers, and, in the meantime, to enhance the competition of industries to be more ecological (the use of co-products, biodegradability), economical (less energy and solvent consumption), and innovative [25]. In agreement with this green extraction approach, unconventional extraction methods such as microwave, ultrasound-assisted extractions, and techniques based on the use of compressed fluids as extracting agents, such as subcritical water extraction (SWE), supercritical fluid extraction (SFE), pressurized fluid extraction (PFE), or accelerated solvent extraction (ASE), are applied to actually separate phenolic compounds [1,26,27,28,29,30,31,32]. Generally, the most common extraction methods for *Vaccinium* phenolic compounds use methanol [9,14,21,33] or acetone [13,18,34,35,36] as extraction agents of bilberry/lingonberry phenolic compounds, but in terms of the utilization for the food and cosmetic industries, ethanol and water are preferred [12,24,32,37,38].

For the quantification and characterization of phenolic compounds from plant extracts, different spectrophotometric and chromatographic methods have been developed. As a spectrophotometric method, Folin–Ciocalteu assay is widely used for determining total phenolics content, vanillin and proanthocyanidin assays have been used to estimate total proanthocyanidins, pH differential methods are used for the quantification of total anthocyanins, and total flavonoid contents can be determined using a colorimetric method based on the complexation of phenolic compounds with Al(III) [24]. Among them, the Folin-Ciocalteu method is the most commonly used. The mentioned spectrophotometric assays give an estimation of the total phenolic contents, while various chromatographic techniques are employed for separation, identification, and quantification of individual phenolic compounds [39]. 

To identify phenolic compounds, the most common technique is high-performance liquid chromatography (HPLC). The HPLC method is also used for the quantitative analysis of phenolic metabolites from different plant extracts [40,41,42,43,44,45,46]. Identification and analysis of phenolic compounds are usually achieved by using a combination of UV–visible spectrophotometry (diode array detector—DAD), mass spectrometry (LC–MS), and nuclear magnetic resonance (NMR) [35,47,48,49]. 

## 4. Potential Pharmaceutical and Biological Activity of Phenolic *Vaccinium* Leaf and Stem Extracts

Bilberry leaves are used as herbal tea and have also been shown to exhibit antibacterial and antioxidant activity [50]. Similarly to bilberry, lingonberry has different biological properties such as antioxidant, antimicrobial, antiadhesive, and anti-inflammatory properties. These benefits are largely attributed to the high content of phenolic compounds in bilberry and lingonberry, compounds that are recognized to have multiple biological activities. In many research papers, it was shown that the antioxidant activity of phenolic compounds is correlated with their health benefits [51,52,53]. Regarding this approach of action of polyphenols as antioxidants in humans, Dangles [3] clarified very well that the cardioprotective effects of phenolic compounds involve their anti-inflammatory rather than their antioxidant properties. Additionally, the issue of limited bioavailability of phenolic compounds and their in vivo metabolism must be taken into consideration. At the same time, in the case of complex mixtures, such as plant extracts, other interfering constituents and non-phenolic antioxidants (vitamins and transition metals) may also be partly responsible for their activities.

### 4.1. Antioxidant Activity of Phenolic Compounds and Vaccinium Extracts

There are three important systems where phenolic compounds can express their antioxidant activity: in plants, in foods, and in humans. The antioxidant properties of these compounds are of particular interest for foods through the inhibition of lipid oxidation, while the protection against oxidative stress is central in plant and human physiology. These properties reflect the reducing properties of phenolic compounds and their ability to interact with metal ions and proteins [2]. In particular, phenolic compounds exert their antioxidant activity by direct scavenging of reactive oxygen species (ROS), inhibition of enzymes involved in oxidative stress, regeneration of other antioxidants (α-tocopherol), chelation of metal ions that are responsible for ROS production, and, finally, stimulation of endogenous antioxidant defense systems.

ROS are generated as a result of partial reduction of oxygen, which leads to the formation of radical oxygen species such as O_2_● (anion superoxide), HO● (hydroxyl radical), NO● (nitric oxide), as well as RO● (oxyl) and ROO● (peroxyl) radicals that are generated during lipid peroxidation (specifically from polyunsaturated fatty acid (PUFA) oxidation) [2,54]. Other reactive species, such as H_2_O_2_ (hydrogen peroxide), ^1^O_2_ (singlet oxygen), O_3_ (ozone), ONOO^−^ (peroxynitrite), HOCl (hypochlorous acid), and HOBr (hypobromous acid), are also ROS that can cause biological damage. Although they are nonradical oxygen species, they are oxidizing agents and/or are easily converted into radicals [55].

The most popular methods for evaluation of the antioxidant activity of phenolic extracts from *Vaccinium* plants are the Folin–Ciocalteu method, DPPH (2,2-diphenyl-1-picrylhydrazyl) radical scavenging method, oxygen radical absorbance capacity (ORAC), cupric ion reducing antioxidant capacity (CUPRAC), ferric ion reducing antioxidant power (FRAP), and Trolox equivalent antioxidant capacity (TEAC) assays [49,50,56,57,58]. The Folin–Ciocalteu method, which measures the ability of a sample to reduce transition metal ions, such as in the complex between sodium phosphomolybdate and phosphotungstate, gives access to the total phenolic content (TPC) in plant extracts using gallic acid as a standard. Since its mechanism is an oxidation/reduction reaction, the Folin–Ciocalteu method can be considered also a method for quantification of the antioxidant capacity. As for the DPPH (2,2-diphenyl-1-picrylhydrazyl) test, it relies on the ability of reducing molecules to transfer an electron or a hydrogen atom to the nitrogen-centered DPPH radical. In the ORAC assay, the peroxyl radical reacts with a fluorescent probe to form a nonfluorescent product, which is quantitated by fluorescence [59,60]. The CUPRAC, FRAP, and TEAC methods are electron transfer -based assays, which changes color when bis(neocuproine)Cu^2+^Cl_2_, Fe^3+^(2,4,6-tripyridyl-s-triazine)_2_Cl_3_, and 2,2′-azinobis(3-ethylbenzothiazoline-6-sulfonic acid) radical cation (ABTS·+) probes are reduced, respectively [1].

Regarding the action of leaves and stems of bilberry and lingonberry as natural antioxidant systems, it has been demonstrated that they are not only rich in phenolic compounds, but also present a significant antioxidant activity highlighted through their capacity to reduce the DPPH radical and transition metal ions in the Folin–Ciocalteu test, and to have a protective effect towards polyunsaturated dietary lipids in the early step of gastric digestion [16]. In the same study, antioxidant activity (DPPH and Folin–Ciocalteu tests) of leaves and stems of bilberry and lingonberry strongly correlated with the phenolic content. 

The antioxidant potential of bilberry leaves (79.30 and 59.58 mM TE/100 g DM in ABTS and FRAP tests) is also stronger than that of fruits (35.34 and 26.81 mM TE/100 g DM in ABTS and FRAP tests as well) [49]. Other researchers investigated the antioxidant activity of water, ethanol, and ethyl acetate extracts from fruits and leaves of bilberry and found that the water extract from leaves of *V. myrtillus* L. has intense antioxidant activity, which is in correlation with the high concentration of total phenols [50]. The antioxidant capacity determined by the ABTS, FRAP, and FIC (ferrous ions chelating) methods of different cultivars and lower taxa of lingonberry leaves extracts were compared, and showed that “Kostromskaja rozovaja” and “Rubin” cultivars, as well as *V. vitis-idaea* var. *leucocarpum*, possessed the greatest reducing, radical scavenging, and chelating activities [61]. 

### 4.2. Cardioprotective Activity

Atherosclerosis, a chronic inflammatory disorder associated with oxidative processes, is the major cause of cardiovascular disease (CVD), including myocardial infarction (MI), heart failure, stroke, and claudication [62]. Other important risk factors of CVD comprise obesity, diabetes, hypertension, high levels of lipids, and uric acid [63]. 

A study on apolipoprotein E-deficient (apo E−/−) mice model of atherosclerosis exhibited that the dietary supplementation with bilberry anthocyanin-rich extract (Antho 50 from FERLUX S.A, Cournon d’Auvergne, France) containing 52% of pure anthocyanins for 2 weeks reduced plasmatic total cholesterol (−20%) and hepatic triglyceride levels (−30% in the liver), whereas the plasma antioxidant capacity remained unchanged [64]. In a following study, these bilberry extracts showed action on the modulation of gene expression involved in angiogenesis in the aortas of apo E−/−mice [65]. 

The potential beneficial effects of bilberry have also been studied on the development of obesity in mice fed with a high-fat diet (HFD) [66]. Mice fed with 5% or 10% (w/w) of whole bilberries in HFD for 3 months had lower glucose and blood pressure levels compared to mice fed HFD alone. Also, the addition of bilberries to HFD was also found to reduce the levels of several parameters of inflammation. The levels of insulin were not affected by the addition of bilberries to HFD. Regarding lingonberry, its fruit juice moderately decreased low-grade inflammation caused by a high-salt diet (a risk for cardiovascular disease) in young rats [67].

Human studies were also reported regarding the cardioprotective activity of bilberry leaves [68]. In a human study of 35 volunteers, Erlund [69] investigated the effects of daily consumption of mixed bilberries (100 g) and nectar containing 50 g crushed lingonberries on well-established risk factors of CVD, such as platelet function, HDL cholesterol, and blood pressure for 2 months. Additionally, the subjects consumed blackcurrant or strawberry puree and cold-pressed chokeberry and raspberry juice on alternating days during the study. No changes were seen in plasma biomarkers of platelet activation, coagulation, or fibrinolysis, but the systolic blood pressure was significantly decreased, and the serum HDL cholesterol concentrations increased. In a recent study, intake of bilberry in conjunction with wholegrain and fish caused significant changes in lipid metabolites in subjects with risk of coronary heart [70].

Cardioprotective actions of products (extracts, juice) of other *Vaccinium* species have been also reported. For example, in a placebo-controlled, double-blind, parallel-arm, human study of 56 healthy adults, Novotny et al. [71] demonstrated that low-calorie cranberry juice, rich in phenolic compounds (173 mg/240 mL juice), lowered factors of cardiovascular disease, including serum triglycerides, serum C-reactive protein, glucose, insulin resistance, and diastolic blood pressure.

### 4.3. Anti-Cancer Activity

Several studies have investigated the effect of bilberry and lingonberry products on cancers. Cell lines, in vitro model systems, and animals or human subjects have been used to test their anti-cancer activity through its antiproliferative and apoptotic effects. 

Procyanidin-rich extract (almost A-type and B-type dimers) from lingonberry [72] and flavonoids and phenolic acid fractions from bilberry fruits [73] are effective in preventing the proliferation of human cervical and colon cancer cells in vitro. The aqueous extract of bilberry press residue after juice production has also shown a stronger inhibitory effect on cell proliferation of three colon cancer cell lines (Caco-2, HT-29, and HCT 116) [38]. This extract was also found to contain the highest total phenolic content (1447 mg gallic acid equivalent (GAE)/100 g of press residue) and total monomeric anthocyanins (458 mg/100 g of press residue), and a correlation has been found between the phenolic concentration of extract and its antiproliferative effect—but it is possible that other compounds (e.g., vitamins) not quantified in this study may also contribute to the antiproliferative effect of bilberry extract. Recently, in a research article published in Scientific Reports, anthocyanin bilberry extract Antho 50 was studied for its apoptotic effect in chronic lymphocytic leukemia cells [74]. This effect was induced through the generation of ROS and was attributed to activation of caspase 3 and down-regulation of UHRF1 and Bcl-2. In one study, other researchers have proven the inhibitory effect of lingonberry methanolic extract and of its anthocyanin-rich and phenolic-rich fractions against apoptosis induced by ischemia-reperfusion [75].

Regarding the carcinogenicity of *Vaccinium* extracts, special attention must be paid to the potential herb–drug interactions that can occur with these extracts and to their toxicity [76]. Relevant experimental studies in these fields are lacking. Nevertheless, a case report was published by Aktas et al. (2011) [77] about the putative risk of bleeding on concomitant intake of bilberry extract and anticoagulants such as warfarin. Unfortunately, there were not enough data to prove the effect of bilberry on anticoagulants and antiplatelet agents. 

### 4.4. Antidiabetic Activity

Hypoglycemia and hyperglycemia are the most common symptoms of diabetes. It is well known that obesity is another important risk factor for the occurrence of type 2 diabetes. Non-alcoholic steatohepatitis (NASH), which represents the accumulation of lipids in the liver, is the highest prediction factor for the occurrence of type 2 diabetes. With reference to the symptoms of diabetes, bilberry and lingonberry products have shown positive effects.

In a human study, dietary supplementation of 31 slightly overweight women with a mixture of lingonberry and bilberry berry products and other berries (163 g of berries daily) for 20 weeks showed a decrease by 23% of alanine aminotransferase level, which is known as a common liver disease marker and an important risk factor of diabetes [78]. No differences were seen in plasma antioxidant capacity measured as ORAC and inflammation marker hs-CRP.

A study conducted in Japan with type 2 diabetic mice found that dietary anthocyanin-rich bilberry extract ameliorates hyperglycemia and insulin sensitivity, and that the effects were due to activation of AMP-activated protein kinase [79]. A standardized bilberry extract Mirtoselect, containing 36% anthocyanins, attenuated hepatic steatosis induced in mice fed a Western-type diet supplemented with 1% cholesterol for 20 weeks [80]. The decrease of macro- and microvesicular hepatocellular lipid accumulation, no increase of hepatic triglyceride levels and reduction of hepatic cholesteryl ester content, hepatic inflammation, and hepatic fibrosis were observed in Mirtoselect-treated mice. In an in vivo human study, the incorporation of commercial lingonberry powder in fat-free yogurt meal supplemented with glucose attenuated the glycemic response of the sugars present in the berries, and it was indicated that the fibers and/or polyphenols present in lingonberries are responsible for this effect [81].

### 4.5. Vision Improvement Properties

For several decades, the consumption of bilberry has been associated with the improvement of human vision in reduced light. A review of 30 clinical trials regarding the action of anthocyanoside-rich extracts of *Vaccinium myrtillus* has been published in order to clarify the positive or negative effects of bilberry on night vision [82]. The results of the studies discussed in this review are somewhat contradictory. Of the studies reviewed, from 12 placebo-controlled trials, 4 randomized controlled trials showed no significant effects on vision in reduced-light conditions. It was suggested that the negative outcome is confounded by several factors, including dose, possible geographical variations in anthocyanoside composition, and choice of subject. The fifth randomized controlled trial and seven non-randomized trials reported positive effects on vision improvement in reduced light. 

Nevertheless, according to the most recent research, beneficial effects of bilberry and lingonberry in vision improvement were evidenced in cell-based in vitro studies. For example, bilberry extract and lingonberry extract and their phenolic constituents (cyanidin, delphinidin, malvidin, *trans*-resveratrol, and procyanidin B2) appear to exert protective effects against retinal damage induced by blue light-emitting diode (LED) light via inhibition of ROS production and activation of pro-apoptotic proteins [83]. Another study by Song et al. [84] showed that a bilberry extract containing 25% total anthocyanins could promote physiological renewal and homeostasis of human corneal limbal epithelial cells (HCLEC). In a study of 23 patients with asymptomatic ocular hypertension, 24 weeks of dietary supplementation with Mirtogenol^®^, a combination of two phenolic extracts from bilberry (Mirtoselect^®^) (standardized to 36% anthocyanins) and French maritime pine bark (Pycnogenol^®^) (standardized to 70% procyanidins), was reported to lower the intraocular pressure up to 24% and improve the diastolic ocular blood flow [85].

### 4.6. Antimicrobial Activity 

Several studies demonstrated the antimicrobial activity of *Vaccinium myrtillus* L. and *Vaccinium vitis idaea* L. Antimicrobial effects of bilberry and lingonberry phenolic extracts from fruits and leaves have been shown especially in the prevention of urinary tract infections [86]. In vitro, lingonberry fruit extracts containing mainly type-A proanthocyanidins may be bactericidal against *Staphylococcus aureus* or inhibit the hemagglutination of *Escherichia coli* [13]. Similar antimicrobial effect of flavonol glycosides, anthocyanins, procyanidins, and flavan-3-ols fractions purified from lingonberry juice was reported against two other pathogens, *Streptococcus mutans* and *Fusobacterium nucleatum* [87]. Other researchers investigated the antibacterial activity of water, ethanol, and ethyl acetate extracts from fruits and leaves of bilberry on strains of *Escherichia coli*, *Enterococcus faecalis,* and *Proteus vulgaris,* and found that all extracts were more effective against *E. faecalis* and *P. vulgaris* [50].

## 5. Conclusions

This synthesis provides information on the potential of the use of leaves and stems of *Vaccinium myrtillus* L. and *Vaccinium vitis-idaea* L. as a promising source of phenolic compounds, so they are suitable for valorization as valuable feedstocks for the production of functional foods or pharmaceuticals with nutritional properties and biological activity against diet-related oxidative stress. To date, most works have focused on the study of the berries of *Vaccinium* species, but on leaves and stems, only sometimes. However, good knowledge of the phenolic compound distribution in the various *Vaccinium* plant tissues can play a key role in guiding their fields of use, and more knowledge is necessary on the biological activity of leaves and stems. The phenolic qualitative analysis of the leaves and stems of *Vaccinium myrtillus* L. and *Vaccinium vitis-idaea* L. first helped to understanding their high activity as antioxidant sources. In human health, this knowledge is of further interest, since an extract enriched in a particular sub-class of phenolic compounds may be selected for a desired biological activity. Moreover, toxicological studies on *Vaccinium* plants are limited and is necessary to be assessed in order to prevent adverse effects and interactions.

## Figures and Tables

**Table 1:** Phenolic compounds identified in aerial parts of *Vaccinium* plants.

Class	Phenolic Compounds	*V. myrtillus*		*V. vitis-idaea*		References
		*Leaves*	*Stems*	*Leaves*	*Stems*	
Catechins	(+)-catechin	x	x	x	x	[10,12,14,15,18,21,22]
	(−)-epicatechin	x	x	x	x	[10,12,14,15,18,21]
	gallocatechin	x	x	-	x	[10,14,15]
	epigallocatechin	x	x	-	x	
Cinchonains	cinchonain I	x	x	x	x	[10,12,14]
	cinchonain II	x	x	x	x	
Proanthocyanidins	B-type dimer	x	x	x	x	[10,12,14,15,18,21]
	B-type trimer	x	x	x	x	
	B-type tetramer	x	x	x	x	[12]
	B-type pentamer	x	x	-	-	
	A- type dimer	x	x	x	x	[8,10,12,14,15,18,21]
	A-type trimer	x	x	x	x	[10,12,14,15,18,21]
	procyanidin A2	x	x	x	x	
	procyanidin B1	x	x	x	x	
	procyanidin B2	x	x	x	x	
Arbutin derivatives	arbutin	-	-	x	x	[12,15,18]
	caffeoyl acetyl arbutin	-	-	x	-	[12,18]
	caffeoyl arbutin	-	-	x	-	[12,14,15,18]
	acetyl arbutin	-	-	x	x	[12,15,18]
Phenolic acids	3,4-dihydroxybenzoic acid	x	x	x	x	[10,12,14,15,18]
	*p*-coumaroyl quinic acid isomers	x	x	x	x	
	*p*-coumaroyl malonic acid *p*-coumaroyl derivatives	x	x	x	x	
	*p*-coumaroyl glucose	x	x	x	x	
	coumaroyl iridoid	x	x	-	-	
	*p*-coumaric acid	x	-	x	-	[14]
	feruloyl quinic acid isomer	x	-	x	-	
	caffeoyl quinic acid isomers	x	-	x	-	
	caffeic acid ethyl ester	x	-	x	-	
	caffeic acid hexoside	x	-	x	-	
Flavonols	quercetin-3-*O*-(4″-HMG)-α-rhamnoside	x	x	x	x	[10,12,14,15,18,21]
	quercetin-3-*O*-galactoside	x	x	x	x	
	quercetin-3-*O*-glucoside	x	x	x	x	
	quercetin-3-*O*-glucuronide	x	x	x	x	
	quercetin-3-*O*-arabinoside	x	x	x	x	
	quercetin-3-*O*-α-rhamnoside	x	x	x	x	
	quercetin-3-*O*-rutinoside	x	x	x	x	
	quercetin	-	-	x	x	[14,15]
	kaempferol	x	-	x	-	[14]
	kaempferol-hexoside	x	-	x	-	[14,21]
	kaempferol-(HMG)-rhamnoside	x	-	x	-	
	kaempferol-*O*-pentoside	x	-	x	-	
	kaempferol-3-glucuronide	x	-	-	-	[18]
Lignans	Lyoniside (9-*O*-β-D-xylopyranosyl(+)lyoniresinol)	-	x	-	-	[23]

x, present; -, not present.
